# Instrumented Compliant Wrist with Proximity and Contact Sensing for Close Robot Interaction Control

**DOI:** 10.3390/s17061384

**Published:** 2017-06-14

**Authors:** Pascal Laferrière, Pierre Payeur

**Affiliations:** School of Electrical Engineering and Computer Science, University of Ottawa, 800 King Edward, Ottawa, ON K1N 6N5, Canada; plafi092@uottawa.ca

**Keywords:** proximity, contact, touch sensing, compliance, pose estimation, surface following, robot control

## Abstract

Compliance has been exploited in various forms in robotic systems to allow rigid mechanisms to come into contact with fragile objects, or with complex shapes that cannot be accurately modeled. Force feedback control has been the classical approach for providing compliance in robotic systems. However, by integrating other forms of instrumentation with compliance into a single device, it is possible to extend close monitoring of nearby objects before and after contact occurs. As a result, safer and smoother robot control can be achieved both while approaching and while touching surfaces. This paper presents the design and extensive experimental evaluation of a versatile, lightweight, and low-cost instrumented compliant wrist mechanism which can be mounted on any rigid robotic manipulator in order to introduce a layer of compliance while providing the controller with extra sensing signals during close interaction with an object’s surface. Arrays of embedded range sensors provide real-time measurements on the position and orientation of surfaces, either located in proximity or in contact with the robot’s end-effector, which permits close guidance of its operation. Calibration procedures are formulated to overcome inter-sensor variability and achieve the highest available resolution. A versatile solution is created by embedding all signal processing, while wireless transmission connects the device to any industrial robot’s controller to support path control. Experimental work demonstrates the device’s physical compliance as well as the stability and accuracy of the device outputs. Primary applications of the proposed instrumented compliant wrist include smooth surface following in manufacturing, inspection, and safe human-robot interaction.

## 1. Introduction

The majority of robotic manipulators currently used in manufacturing are designed to meet very high precision and repeatability requirements, which influence the design of their mechanical structures, and generally leads to very rigid frames and tightly tuned control loops. However, such constraints become a limitation when dealing with unstructured environments. Modern robots must operate in more complex scenarios and promptly adapt their behavior with only limited a priori knowledge of the actual workspace configuration. Examples of such challenging workspaces are those where objects are not necessarily fixed in any particular orientation or position, or where objects may have imprecisely defined properties such as compressibility or mechanical degrees of freedom. Other situations that truly pose challenges, beyond safety considerations, from a robot control perspective are human-robot interactions where human beings need to either come into contact or work in close proximity to a robot.

On the other hand, rigid industrial robots have been prominent for decades and continue to find ever increasing adoption in the manufacturing sector. They represent a significant investment in terms of design and fabrication costs. It is therefore a promising path to conduct research to determine how existing robotic platforms could be further leveraged and enhanced to meet the requirements of modern applications. Adaptation of the robotic system to these conditions should be achieved without making significant changes to the robots’ fundamental structure, while introducing a level of self-adaptation and response to a transforming environment. Taking inspiration from how humans interact with their environment, compliance, which allows for smooth contact with objects and reduced risk of damage or injury, was identified as a key aspect required for adaptive and responsive robotic interaction control. However, compliance tends to affect the resulting pose of a robot and provisions are required to sense these changes in order to maintain accurate positioning. Doing so effectively provides a complex sense of touch to a robotic manipulator. Humans, along with their sense of touch, also efficiently combine visual information with force and tactile data to help with pose and shape estimation, leading to accurate surface following or object manipulation, which represent common tasks required of robots. However, although computer vision has been widely researched and integrated into robotic platforms, it tends to require a great deal of computational power in order to extract any meaningful and accurate information about an object’s position or orientation, while also being severely impacted by occlusions and other external factors. To prevent the need for sophisticated algorithms and vision sensors, simpler alternatives are studied to extract the most pertinent information about an object’s location and orientation, while operating in proximity to or in contact with it. With such information, a proper approach plan can be formulated prior to initial contact with an object, thereby reducing the risks of damage resulting from inappropriate contact.

This paper, which consists of a significantly extended version of [1], details the design, development and extensive experimental characterization and performance evaluation of a novel sensing device. The latter is capable of simultaneously providing compliance to a rigid robotic structure, while measuring the relative position and orientation of objects located in its proximity, as well as the surface coordinates of objects in direct physical contact with the device. The sensing device is referred to as an instrumented compliant wrist. It is meant to serve as an un-actuated end-effector attachment that can be mounted on a variety of rigid industrial robotic arms in order to provide the existing robot controller with extra real-time sensory information to safely operate either in close proximity with a target object or maneuver while in contact with said object. It supports fine motion guidance in applications such as smooth surface following or interaction with sensitive objects. Following a survey of the literature in Section 2, the broader research that led to the design of the device is introduced in Section 3 before the system’s mechanical and sensing layer structures are presented in Section 4. Section 5 reports on an extensive series of tests conducted with a physical prototype of the compliant wrist to characterize its properties and validate its operation while being inserted in the closed loop control of a manipulator. Finally, Section 6 derives some general conclusions.

## 2. Literature Review

One key advantage that biological systems have over many robotic technologies, beyond their wide range of sensing capabilities, is their intrinsic elastic properties and material flexibility. This is often referred to as compliance in robotic systems. Compliance provides an adaptable interface between the environment and the robot that can relax some of the strict constraints often seen in complex motion planning techniques. Compliant manipulators have been designed with intrinsic compliance either in the form of flexible links or by incorporating compliant structures directly into the connecting joints. In [2] a manipulator makes use of series of elastic actuators [3] that incorporate a degree of compliance into the joints by making use of springs. In the context of force control, elasticity influences the control scheme of a robot. Much like humans who can feel forces being applied to the body but lacking the means of precisely measuring those forces, simply being aware of these forces by inferring them nevertheless allows for the ability to react to them when sensed. The concept of making touch a primary source of information during motion guidance is analogous to how humans are able to interact with their environment when their vision is impaired. The work of Bach-y-Rita and Kercel [4] provides useful insights as to how the human brain can make use of one type of sensory information and effectively translate it into another form. The Obrero manipulator [5] was inspired by how humans manipulate objects, favoring sensing of their environment via multiple modalities over precision. However, the development of robotic systems which are able to take advantage of these innovative ideas can be a costly endeavor, mostly because of the required modifications to existing mechanisms [6]. With the large and ever growing supply of industrial robots in operation, innovative ways to apply the concepts used in compliant manipulators, but without incurring major physical transformations and massive investments, are to be favored. The proposed compliant wrist design documented in this paper aims at bridging this gap.

Sensor based control of robots is recognized to improve flexibility, but also speed and precision, in uncalibrated working environments. Force sensors have been extensively used for classical constrained hybrid force/position control [7,8,9] where force control is included for tasks that involve contact with surfaces. Alternatively, tactile sensors can support the determination of physical properties of objects such as shape, orientation, curvature, and allow for slippage detection [10,11]. However, these sensors are only of use when there is a physical interaction between the robot and the objects or surfaces. In addition, their bandwidth is limited by the mechanical constraints imposed on sensors during the interaction. Visual servoing solutions [12,13,14,15] have been successfully applied to extract features over objects before and during manipulation tasks in order to achieve close guidance of a robot, even generating some forms of visual compliance [16]. In contrast to force/tactile control, vision-based control systems require no contact with the object, allow for non-contact measurements of the environment and do not require formal geometric models of the objects submitted to interaction. Using vision sensors can also prevent loss of contact with the surface. As a result, vision measurements have been combined with force/tactile measurements to form hybrid visual/force control schemes [13,17]. However the extraction of useful information from cameras offers important challenges [18]. Computationally demanding algorithms for reliable feature extraction and for pose estimation, and complicated calibration processes are required. A more ideal sensor should be able to readily, and efficiently, provide the desired information about an object or surface of interest located either in proximity or in contact with a robot end-effector, but without requiring special purpose algorithms, and be operating independently from the specific architecture of the robot it is mounted on.

Unlike fully compliant manipulators, as described above, compliant wrists providing subsets of the desired characteristics have been investigated in the literature. Compliant wrists are devices mounted at the extremity of a manipulator robot, in place of or before the specialized tool or gripper, and are meant to provide a level of compliance to the robot but only locally, that is where interaction with objects or surfaces usually takes place, at the end-effector. An initial design was presented in [19] for improving the accuracy of industrial robots in manufacturing applications. The compliant wrist was instrumented to provide position information to the robot controller as small positional deviations in the wrist. Another compliant wrist sensor [20,21] made use of capacitive sensing properties to measure bending moments in 3D, as well as force in the direction perpendicular to the interaction surface. The capacitive sensors were implemented as circuit boards with 6 mm thick natural rubber material to provide compliance. The limited thickness of the compliant layer however provided only a very limited range of movement and therefore limited the applications of the device. A compliant wrist designed to suit tele-operated robot applications was introduced in [22]. The device, inspired by the Stewart platform concept, supported six degrees of freedom (DOF) through the use of linear compression springs in place of actuators. Optical encoders measured compression distances, which were converted into force estimates. In [23], a compliant wrist was designed based on passive compliance analysis. The wrist also formed a parallel linkage chain but was simplified to only three DOFs. It used linear potentiometers to produce estimation of two rotations and one translation. Its kinematic model however resolved to a complex implementation due to the movement of the joints and position of attachment points. Elastic actuators have also been incorporated into compliant wrist designs such as in [24] to create physical rehabilitation devices. Embedded sensors measure the force or deflection of the joints, and enable the use of force-feedback control. Paul et al. [25,26] introduced a compliant wrist structure that consists of two plates separated by a compliant, damped rubber structure to provide passive compliance and is equipped with a sensing mechanism to measure the deflections of the 6 DOFs allowed by the compliance. The sensing mechanism is composed of a serial linkage chain with potentiometers at each joint for angular measurements. Through the kinematic model of the assembly, the position of the moveable plate, not directly attached to the robot, can be inferred. Moreover, by carefully selecting the rubber compliant components, the stiffness of the structure along each DOF could be estimated. This fact helps reinforce the idea that direct force sensing is not essential for robot control, as this information can be obtained by inferring equations or even approximations depending on the task at hand. Another compliant wrist was designed for performing surface exploration tasks with the goal of extracting geometric features of the surface being contacted [11,27]. This compliant wrist can provide gross position and orientation estimations as well as finer geometric surface profile information from a tactile sensing array appended on its compliant contacting plate. The latter however allowed very little displacement and angular movement due to the attachment of rotary potentiometers to sliding shafts, which highly constrained the motion.

## 3. Context and Design Requirements

**Figure 1:**
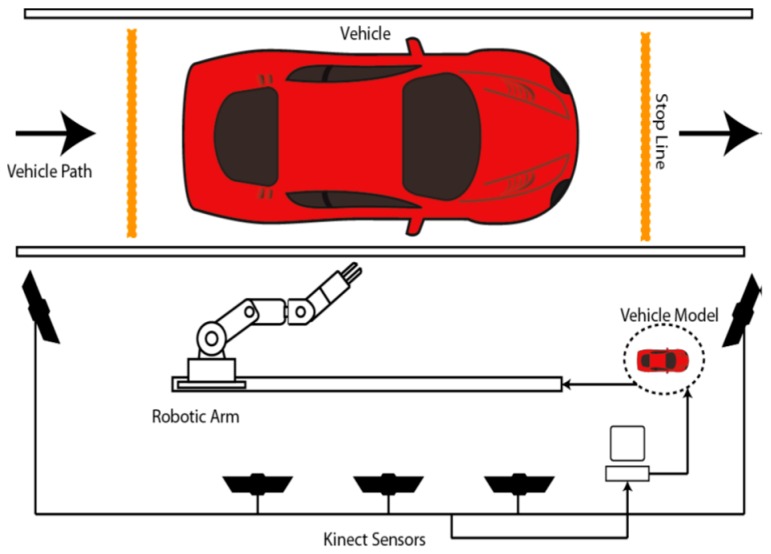
Conceptual automotive inspection system layout.

The instrumented compliant wrist was designed in the context of a larger initiative in which a standard robotic manipulator required adaptation to operate as a robotic system with multi-modal sensing capabilities in order to perform rapid and safe automotive vehicle screening [28]. The scope of the research was that of safely collecting minute amounts of particles over automotive vehicle body parts with a manipulator robot in order to detect traces of dangerous or prohibited substances, as often required to ensure the security of public institutions and critical infrastructures. Adaptive robotic technologies driven by multi-modal sensing systems were developed to fully automate the particles collection process, which is performed by swiping a special piece of fabric over the surface. Compliance was found to be an absolute requirement to ensure the integrity of the vehicles, to provide capabilities to cope with highly variable and imprecisely defined models of the shape of automotive vehicle bodies, while also allowing for ease of integration with commercial, off-the-shelf, precise and fast manipulator robots. The instrumented compliant wrist solution provides the necessary supporting technology to automatically adjust the particles collection procedure to the diversity of shapes and sizes that characterize vehicles of various types, brands and categories. The overall solution is conceptually depicted in Figure 1 and is comprised of several components, some of which directly influenced the design of the compliant wrist.

**Figure 2:**
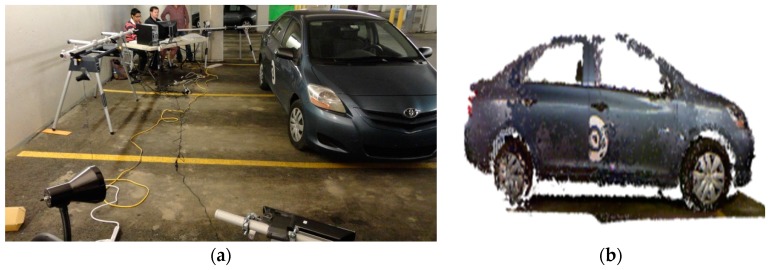
Inspection system prototype with: (**a**) network of Kinect sensors acquiring a rough model of the entire length of vehicle; and (**b**) approximate 3D model of vehicle’s lateral body panels.

In order to initiate the screening process, while ensuring inspection times restrained to only a few minutes, external vision sensors were used to support various parts of the task, such as detecting the arrival of a vehicle, recognizing its class and rough shape, and rapidly acquiring an approximate three dimensional model of its surface [29]. For that purpose, a calibrated network of five Kinect sensors was incorporated into the system, as shown in Figure 2a, allowing for a fast but approximate 3D reconstruction of the vehicle body parts. The rough 3D vehicle model was used for planning an initial trajectory for the robotic manipulator to get in proximity to the surfaces of the vehicle within preselected areas of interest. That is, the approximate 3D model rendered from the Kinect sensors network only supports a rough path planning for the manipulator with the objective to ensure complete coverage of the regions of interest over which particles must be collected. In fact, given that fast but low accuracy Kinect sensors were selected to satisfy tight inspection time constraints, visual data, both color and depth, could not be acquired and merged with sufficient resolution to guarantee accurate and safe surface following, as depicted in Figure 2b. Moreover, vision sensors being located at about 2 m from the vehicle, and with the manipulator robot being inserted in between the vision sensors and the vehicle, as shown in Figure 1, the vision stage suffered from temporary occlusions as the robotic manipulator moved throughout the scene, resulting in a high degree of uncertainty in the manipulator’s control. In order to precisely control the motion of a robotic manipulator in close proximity to and while in contact with a vehicle (to collect particles), and without the possibility to finely model the vehicle’s surface to support the safe usage of a rigid manipulator, the proposed instrumented compliant wrist was designed to provide the robot controller with supplementary and sufficiently accurate information in order to safely operate in close proximity with the vehicle and also maneuver itself while in contact with the vehicle body parts.

In an effort to provide such information as well as to address the limitations of current technologies previously mentioned, a dual layer sensing configuration was conceived along with a physical mechanism meant to integrate a degree of compliance. A first sensing layer embedded in the compliant wrist allows for the detection of objects in the vicinity of the manipulator’s end-effector in real time. Infrared range sensors with a higher resolution on distance measurements than those integrated in Kinect sensors lead to a higher degree of precision and confidence during motion control. The infrared range sensors are arranged in a specific pattern to provide the added functionality of estimating the orientation and position of objects or surfaces in proximity to the robot end-effector. A second sensing layer precisely estimates the amount of deflection imposed onto the compliant wrist plate when contact is achieved with a surface, that is during physical interaction with the environment, as happens for particles collection or any application of surface following. Again, the sensors are arranged in a specific pattern to allow for estimating the relative orientation of a surface with which the compliant wrist is interacting.

The physical structure of the device is designed to offer a significant amount of compliance to act as the contact interface between a rigid manipulator robot and the environment. It serves as a physical buffer, providing the control system with additional time to respond to changes in sensory data, and simplifying the control scheme during transitions between a state of free motion (non-contact) and constrained motion (contact), therefore eliminating the need for formal hybrid position and force control.

Although many benefits can be observed for the robotic manipulator while operating with the additional compliance and sensing mechanisms embedded in the compliant wrist, the information necessary for localizing objects or regions of interest within the workspace still needs to be supplied by the peripheral vision stage to the robot controller. The global solution developed therefore relies on three multi-modal sensing levels, from peripheral imaging for rough path planning with the Kinect sensors network, to proximity interaction monitoring via the first infrared range sensors array, and finally to in-contact sensing via the second array of embedded infrared range sensors. Object recognition and localization, as well as specific path planning remain beyond the scope of this paper, and are addressed in separate publications [29,30].

## 4. Instrumented Compliant Wrist Design

Building upon the principle of multiple modality sensors, a design is proposed that provides feedback to the robot arm controller both while the end-effector is approaching a surface, and after contact is achieved. This not only provides additional information to the robot but also allows it to take advantage of the pre-contact information, increasing safety during navigation and fulfilling the gap of information available from peripheral vision sensors that may be occluded or lacking accuracy [30]. Such peripheral sensors, like the vision stage using Kinect sensors described in Section 3, can easily become occluded from parts of the scene when the manipulator robot passes in front of the cameras, as can be observed in the configuration depicted in Figure 1. Such a temporary loss of visual contact with the surface to interact with is compensated by the proximity of the embedded sensors on the compliant wrist, therefore leading to safer and more accurate operation.

### 4.1. Mechanical System Design

**Figure 3:**
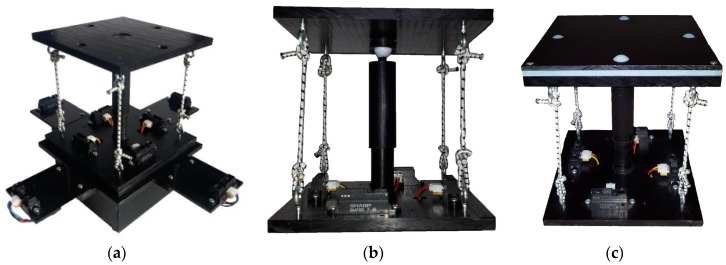
(**a**) Mechanical assembly of compliant wrist prototype; (**b**) detailed view of compliance stage with compression/tension mechanisms and internal infrared range sensor array; and (**c**) wrist with additional contacting layer.

The compliant wrist assembly consists of two plates, as shown in Figure 3a, separated by components, including springs and elastic ropes, which allow for deflection of the upper compliant plate with respect to the bottom static plate when external forces are applied. Instrumentation capable of dynamically measuring this deflection is embedded within the wrist assembly, providing a sense of touch to the device. Additional instrumentation is added to the periphery of the bottom static plate to measure the location of an object’s surface before it comes into contact with the upper compliant plate, providing the proximity detection capability of the device. The combination of these two sensory layers provides the necessary measurements for fine tuning the movements of the robot arm while maneuvering in close proximity to the surface with which it is meant to interact but before contact occurs, as well as dynamically adapting the end-effector’s configuration to conform to the surface’s position and orientation after contact occurs. Figure 3a presents the primary mechanical components of the device. The enclosure at the base of the wrist houses an embedded microcontroller, wireless communications module, and battery-based power source. The mechanism which provides passive compliance sits above the electronics enclosure and is shown in more details in Figure 3b. As discussed previously, compliance was determined to be an essential component to achieve safe and accurate particles collection during the automotive screening process. Passive compliance is provided by a mechanism that combines compression and tension ropes that apply forces on the upper plate to maintain an equilibrium state when no external forces are applied to it. Passive compliance provides a simpler and cost-effective alternative to active compliance, as found in previous designs in the literature, to achieve the desired capabilities. Under influence from external forces, the upper plate rotates about its pivot point centered under the plate and can compress toward the bottom static plate thanks to a compression spring inserted in the central shaft. The method used to position the center compression spring, namely a hollow shaft allowing for translational motion between the two plates, ensures that the position of the moveable plate remains centered on the translational axis during its rotational movements. This contributes to simplify the equations required to model the mechanism and extract the relative transformations between the two plates, which serve to control the manipulator. Four tension elastic ropes, one attached between each of the four corners of the two plates keep the upper compliant plate in contact with its pivot point and add stiffness to the assembly. The combination of opposing forces from the compression spring and tension ropes maintain the wrist in an equilibrium and tensioned state, while allowing one degree of freedom in translation in the direction of the main shaft. Finally, a Teflon (polytetrafluoroethylene, PTFE) ball is inserted at the tip of the upper half of the slide shaft, ensuring a low friction point of contact between the upper compliant plate and the shaft, giving the wrist its two rotational degrees of freedom. An additional contacting layer can be added to the compliant plate to facilitate the gliding of the device along a surface during the considered inspection task. As shown in the upper part of Figure 3c, the layer contains four white 0.25 inch diameter PTFE balls that allow the compliant plate to roll over surfaces with which it comes into contact.

**Figure 4:**
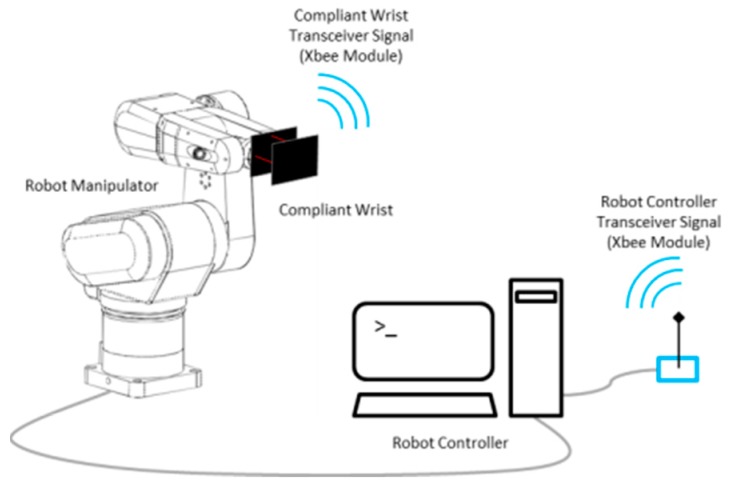
Compliant wrist assembly mounted on a manipulator and wireless communication with robot controller.

**Figure 5:**
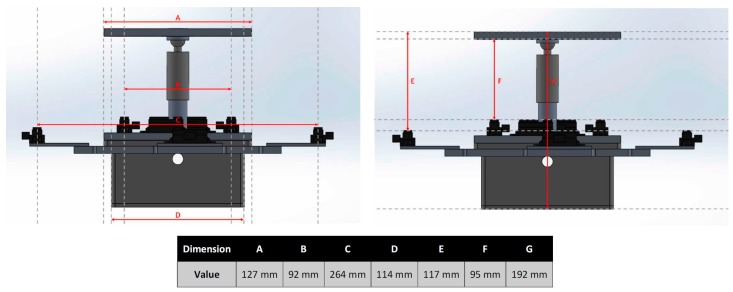
Instrumented compliance wrist physical dimensions.

A USB connection port provides access to the microcontroller for reprogramming purposes, or tethered operation to provide power to the device for extended periods of time. The bottom enclosure, visible in Figure 3a, also forms a bracket that can be customized to the end-effector footprint of virtually any manipulator robot, providing an effective way to mount the compliant wrist as any other robot tool and integrate it with any robot controller, as shown in Figure 4. Embedded wireless communication ensures that all information generated by the compliant wrist is delivered to the robot controller. The communication channel is bidirectional allowing also the robot controller to make data requests as necessary. This information, coupled with the state information of the robot, is used by the robot controller within the implemented trajectory planning algorithms to direct the motion of the robot. Wireless communications and having the device primarily powered by batteries was a design choice meant to increase the compliant wrist module’s versatility. These two key features allow for the device to be self-contained as no physical connections to external devices are required during operation. This further facilitates integration on industrial manipulators as running cables along the structure, and avoiding pinching points is not required. Figure 5 provides supplementary details about the physical size of the compliant wrist prototype. The height of the device is mainly conditioned by the operational range of the infrared sensors contained within the compliant wrist, while the width of the device is influenced by the location of the peripheral array of infrared sensors that must be positioned sufficiently far from the compliant plate to prevent the latter occluding the surface under observation in front of the robot’s end-effector.

One known limitation of the current design of the compliant wrist mechanism is that it is possible for the upper compliant plate to slightly rotate about the longitudinal axis of the central shaft while the sensing layer cannot measure this rotation. One way to address this limitation would consist of integrating an extra rotary sensor with color stripes under the compliant plate coupled with an infrared sensor to count the angular deviation. However, local roll around the pointing direction of the compliant wrist, or robot tool, does not significantly affect the adjustment of the device to match the position and orientation of an external surface the robot is interacting with, especially for surface following tasks where the roll parameter is not the most critical. This degree of freedom in rotation can therefore be neglected for most of these applications, and high precision measurement on this extra degree of freedom did not appear essential and was therefore not implemented.

**Figure 6:**
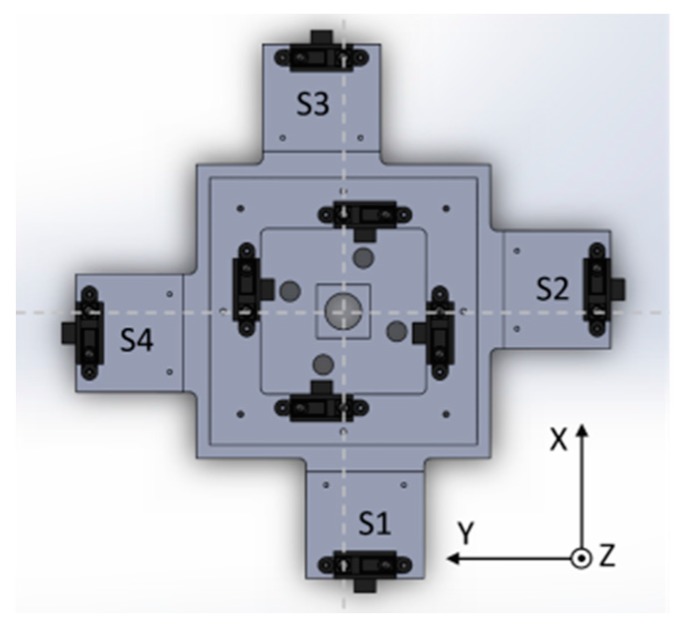
Internal and external range sensors arrangement and compliant wrist’s reference frame.

Four analog infrared (IR) range sensors are mounted to the bottom plate and positioned in such a way as to allow for direct measurement of the distance between the sensors and the inside face of the movable upper plate, as shown in Figure 6. These are referred to as the internal, or contact, sensors. Four additional IR sensors located at the outermost periphery act as the proximity sensory layer and measure distance to closest objects in front of the compliant wrist, that is, on surfaces present in the surroundings of the compliant plate and in front of the end-effector. These are referred to as the external, or proximity, sensors. Figure 6 provides a top down view of the positioning of all eight range sensors on the compliant wrist, with the external sensors array marked as S1 to S4.

### 4.2. Embedded Instrumentation

**Figure 7:**
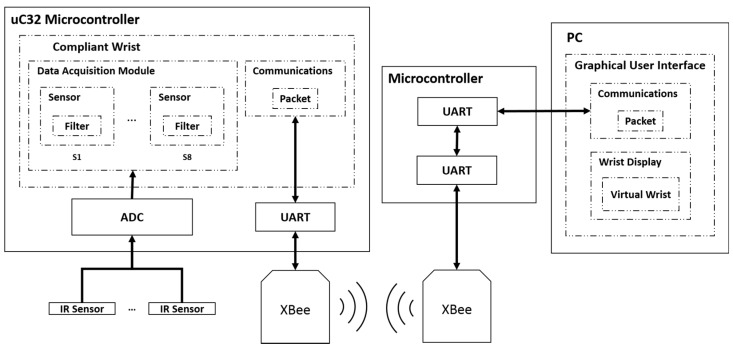
Compliant wrist sensing and communications system diagram.

The eight infrared range sensors are the key components for the instrumentation of the compliant wrist module. They allow for the detection of objects in proximity to the device as well as an indirect means of detecting physical contact between the device and its environment by measuring deflections of the movable (upper) plate interface. Raw data collection, processing and interpretation are entirely performed on-board via an embedded Microchip PIC32MX340F512H microcontroller capable of operating at 80 MHz. Eight analog Sharp GP2Y0A41SK0F infrared range sensors collect distance measurements at up to 80 Hz over a range of 4 to 30 cm. The same model of IR sensor is used for both the internal and external sensing layers. Accurate distance measurements are achieved over the range of 30 mm from the upper compliant plate and up to 300 mm, which is adequate for measuring the position and relative orientation of surfaces in the proximity of the end-effector, complementing well the peripheral vision stage during close approach of the robot to the surface with which it must interact. Wireless communications are ensured via XBee S1 radio modules. One node is embedded in the compliant wrist, and a second node is connected via a USB port to the robot controller, or controlling computer, as shown in Figure 7.

Wireless transmission rates are executed at a speed of 250 kbps while transfers of data between the radio module and peripherals can be performed over a range of speeds depending on the peripheral requirements. The particular nodes selected are classified as being low power, which extends battery life, and support a communication range of up to 30 m, which is sufficient to allow operation of the compliant wrist on a majority of manipulator robots. Throughout our extensive experimental tests, no loss of communication between the compliant wrist and the robot controller port was observed, thus resulting in a reliable connection while operating within a 3 m × 2 m × 2 m workspace. Given the nature of the tasks considered during the phase of development, XBee nodes operated in the broadcast mode and no special consideration was given to secure the communication channels.

The compliant wrist data acquisition stage developed to gather data from the two layers of range sensors, process the measurements, and extract meaningful information to a robot controller, is illustrated in Figure 7, where solid lines represent physical hardware, either embedded in the compliant wrist or located in the robot control box or controlling computer, and dashed lines represent software blocks. The embedded microcontroller is responsible for computing the distances corresponding to the raw voltage measurements from each IR range sensor, and converting them to actual estimates for each of the three degrees of freedom of the wrist, namely one translation along the Z axis, as depicted in Figure 6, and two rotations about the X and Y axes, from each set of four sensors (internal or external respectively). The physical configuration of the sensors embedded in the wrist, as shown in Figure 6, is encoded in the microcontroller, which allows for recovering the relative 3D affine transformation in between the bottom static plate and the upper compliant plate when the device is in contact with a surface, or in between the bottom static plate and the surface of an object in close proximity to the end-effector prior to contact. A set of four separate normal vectors associated to the orientation of the surface in proximity to the wrist is also extracted when operating with the external array of sensors. The resulting kinematic information linking the compliant plate, or object surface, to the robot end-effector’s reference frame, or tool frame, provides a compact representation of the effect of the interaction, upon which the manipulator can rely to finely adjust its configuration in order to match the end-effector with the located surface position and orientation, and proceed with accurate surface following.

### 4.3. Sensors Calibration

**Figure 8:**
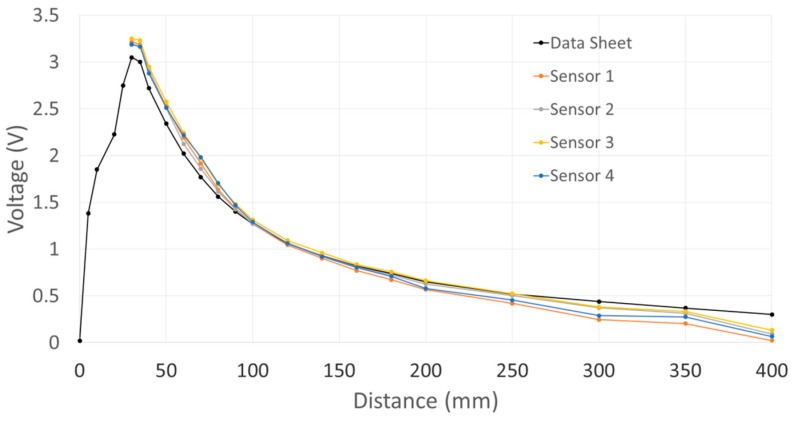
Characteristic response of individual IR range sensors.

**Figure 9:**
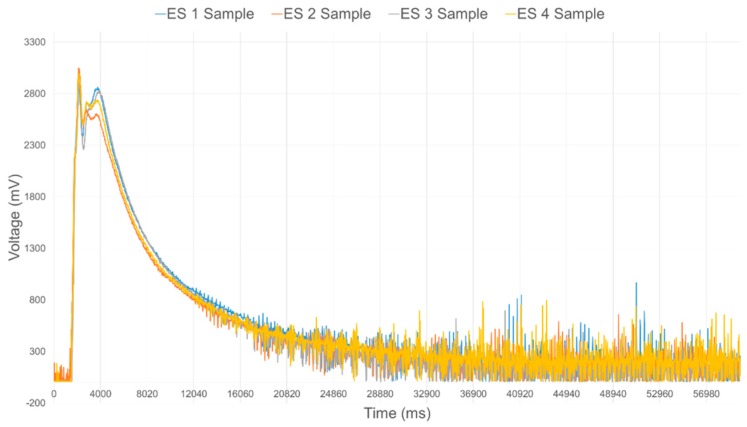
Raw response of four individual IR range sensors over the full range of operation.

**Figure 10:**
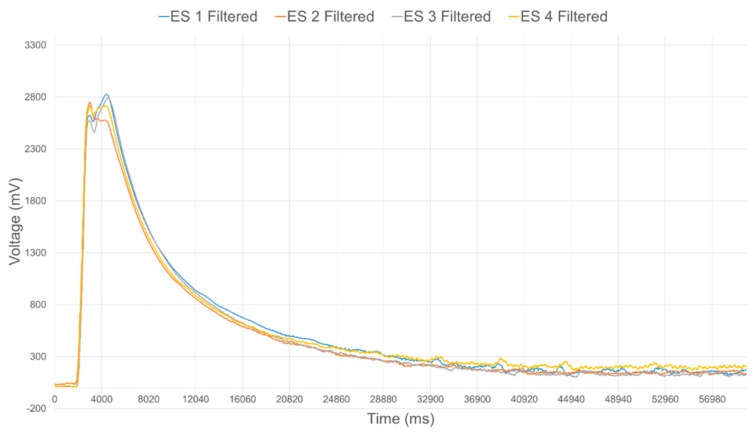
Filtered response of four individual IR range sensors over the full range of operation.

The data acquisition module provides the interface to each of the IR range sensors, but most importantly processes the data to filter noise and calibrate the response from each individual sensor prior to performing data fusion. In order to effectively integrate the IR sensors into the compliant wrist system, an extensive experimental study of their operational characteristics was conducted. The characteristic output of these IR sensors exhibit a nonlinear relationship to the physical distance, as shown in Figure 8. Figure 9 shows the raw voltage signal of four IR range sensors when measuring the distance to an object located in front of the sensors while it is moving away from those sensors. Increasing noise is visible as the surface reaches furthest points away from the sensors. Moving average filtering of the raw signals with a window length of 33 samples is therefore performed on-board the compliant wrist, which results in the voltage response shown in Figure 10 from the same sensors and over the same range of distances.

**Figure 11:**
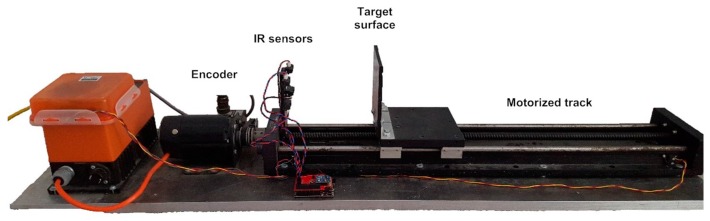
Embedded range sensors calibration procedure.

Moreover, as also observed in Figure 8 for four sensors, different IR range sensors typically provide significantly different raw voltage measurements when operating over identical distances to a surface, and independently from the reflectance characteristics of that surface. It was determined experimentally that the difference between sensors is not a simple constant offset. In order to achieve the uniformity among sensor readings that is necessary to perform data fusion allowing for the determination of accurate affine transformations, a formal calibration procedure was developed to ensure consistency and to increase accuracy of the compliant wrist measurements, both for proximity and contact interactions. In essence, this procedure entails fitting the native response of each IR sensor to a desired response curve, and applying the necessary corrections. For that purpose, voltage offsets are experimentally determined over the entire range of operation and for every individual sensor prior to embedding the devices in the compliant wrist. The calibration procedure involves mounting IR range sensors on a motorized track equipped with an accurate encoder and a flat target surface, as shown in Figure 11. The target surface is successively positioned at 18 calibration distances spread over the range of operation of the IR range sensors, and the native output of each IR sensor is recorded, to be compared with the ground truth distance measured from the track encoder.

For every calibration point, and for every individual sensor, the output is offset until the distance matches the ground truth distance. For each sensor, a series of 18 voltage offset values is encoded in the model of the compliant wrist, which is stored in the microcontroller persistent memory, allowing for the calibration to be easily updated upon replacement of defective sensors, without the need to reprogram the microcontroller. Detailed results obtained following the calibration procedure will be presented in Section 5.1.

### 4.4. Kinematic Representation

The physical design of the proposed compliant wrist provides for a simple kinematic representation of the device with three DOFs, namely two rotational DOFs and one translational DOF. As the device employs two independent sets of four IR sensors, both sets of sensors operate in the same fashion and are capable of generating similar distance information from their respective anchor points. However, the way in which the distance information is interpreted is dependent on the sensory layer considered as well as the intended application. The internal sensors are used for measuring the deflection of the movable contact plate while the external sensors are monitoring a surface or an object in the environment, in proximity to the compliant wrist but without any contact being involved. They serve to estimate the relative pose of the surface with respect to the compliant wrist. Moreover, when dealing with the external sensor array, any surface shape can be encountered. Normal vectors meant to further describe the general surface characteristics of the encountered objects are also estimated from the measured distances to refine the object’s shape description and its relative orientation [31]. For the internal sensors, since the surface of the movable plate is assumed to be uniformly planar (by design), the representation involves only two rotations and one translation to completely characterize the detected displacement of the compliant plate.

Referring to Figure 6 where the physical configuration of all eight sensors (internal and external arrays) is defined with respect to the local compliant wrist 3D reference frame, the extraction process for the relative rotation about the X and Y axes, and translation along the Z axis, as well as for the normal vectors to an external surface, relies solely on distance measurements obtained from the IR range sensors and the position of each sensor on the compliant wrist relative to the assigned origin. The reference frame associated with the compliant wrist has its origin located in the center of the sliding shaft and stationary plate.

When operating in proximity mode, the IR sensors can potentially generate measurements at various points on a surface that can be non-planar. Given that four measurements are available and that a planar surface can be defined from a group of three points only, four normal vectors corresponding to four distinct sub-planes in 3D can be estimated. Normal vectors to the observed surface are obtained from the cross product of two vectors generated from the differences between two sensor pairs, as generally defined in Equation (1):

(1) $$\vec{N_{n}}\;=\;\vec{S_{n}S_{n+1}}\;\times \;\vec{S_{n}S_{n-1}}$$

where *S_n_* corresponds to any of the four external sensors (from S1 to S4), *S_n+_*_1_ corresponds to the next sensor in the group, and *S_n_*_−1_ corresponds to the previous sensor. Combining the resulting four normal vectors with the original distance measurements of the sensors allows the estimation of the overall surface orientation and curvature to be refined by creating multi-faceted representations of detected objects. Calculating this on-board the compliant wrist allows for the possibility of using the external sensor layer for approximate surface shape representation in the event that information pertaining to the description of the area in front of the robot in question was not previously acquired with the peripheral vision system, either due to lack of sensor data, limited resolution, or possible occlusion of the workspace.

**Figure 12:**
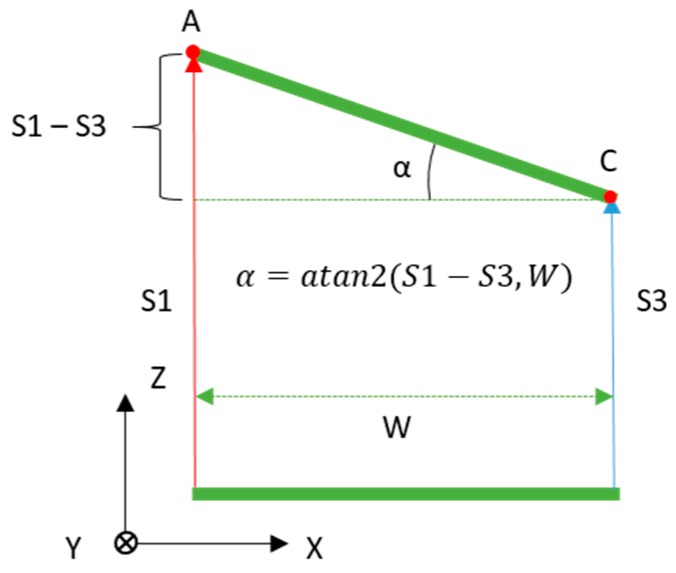
Estimation of relative surface rotation, about Y axis, with respect to compliant wrist base plate.

For each of the two rotations, the angle is calculated from the distance measurements of two IR range sensors that are aligned along a particular axis of the compliant wrist reference frame. The rotation about the X axis (R_x_) is extracted from the distances measured by the two sensors aligned along the Y axis, that is S2 and S4, and similarly the rotation about the Y axis (R_y_) is extracted from the distances measured by the two sensors aligned along the X axis, that is S1 and S3. The measured distances extracted from the sensors correspond to the Z component of the vectors associated with each sensor. Under the assumption that all IR sensors measure along parallel directions in 3D space, which is imposed by construction, taking the arctangent of a right angle triangle whose vertical side is the difference in distance measurements of the two sensors on that axis with a base whose length is equal to the distance that separates the two sensors, the desired angle is obtained. Figure 12 illustrates how the rotation angle about the Y axis is calculated on-board the compliant wrist from two distances measured from IR range sensors aligned along the X axis, assuming an in-between sensor distance, *W*.

The process is defined in Equations (2) and (3), respectively for rotations, R_x_ and R_y_, about the X and Y axes:

(2) $$R_{X}\;=\;\beta \;=\;atan2\left(S4_{\left(Z\right)}-S2_{\left(Z\right)},W\right)$$

(3) $$R_{Y}\;=\;\alpha \;=\;atan2\left(S1_{\left(Z\right)}-S3_{\left(Z\right)},W\right)$$

where *S*#_(Z)_ indicates the distance measurement from a given sensor. These equations are valid regardless of which sensor array is being used, either the internal or the external. Only the separation, *W*, takes on different values depending on the physical locations of the sensors with respect to the reference frame. In our prototype, *W*, corresponds to 92 mm for the internal array, and 264 mm for the external one, as illustrated in Figure 5.

The third DOF of the compliant wrist structure is translational in nature and occurs along the Z axis. This translation along the Z axis (T_z_) is also estimated from distance measurements generated by the IR range sensors, considering the average of the 4 distances reported by the sensors, either internal or external. In the case of the internal sensors, this average distance value corresponds to the distance between the centers of the lower static and upper compliant plates. For external sensors, this calculation provides the distance to an object in front of the compliant wrist, under the assumption that the surface observed is planar. As a result, the translation estimate slightly increases in accuracy with the actual planarity of the object. The calculation is carried out as defined in Equation (4):

(4) $$T_{Z}\;=\;\left(S1_{\left(Z\right)}+S2_{\left(Z\right)}+S3_{\left(Z\right)}+S4_{\left(Z\right)}\right)/4$$

**Figure 13:**
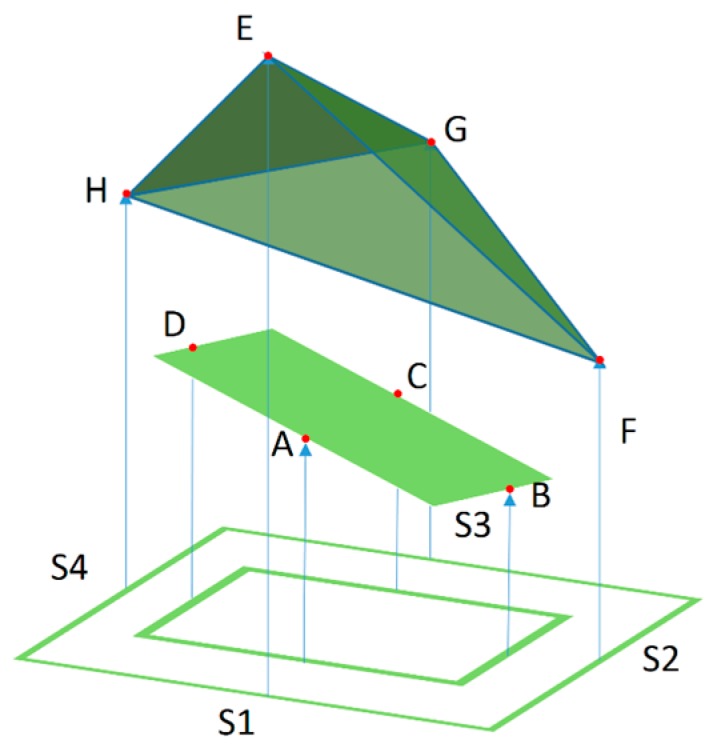
Compliant wrist kinematic configuration estimation.

Given that the compliant wrist effectively acts as an additional link to a manipulator robot, encoding the relative position and orientation of the compliant plate, or proximal surface, as an affine transformation matrix is an efficient way to actively integrate the device with the robot controller. The parameters of the matrix depend on the four distance measurements, represented as A, B, C, and D in Figure 13 for the internal array of sensors; or as E, F, G and H for the external array. As mentioned previously the external sensors will not necessarily be dealing with object surfaces that are devoid of curvature or deformations. In such cases the distances obtained can lead to a surface representation such as the one shown on the upper part of Figure 13, which can be detected through the determination of the four normal surface vectors, as defined in Equation (1).

The homogeneous transformation matrix, Q_wrist/effector_, which describes the pose of the compliant wrist’s compliant plate (which involves only the four internal IR sensors) with respect to the point of attachment onto the robot end-effector is estimated from two components. The first is a pure translation matrix in the Z direction which describes the offset between the point of attachment of the end-effector and the origin of the compliant wrist set by the mounting position of the infrared sensors. The second is the matrix which describes the relative position of the compliant wrist’s compliant plate with respect to the infrared sensors. The formulation is summarized in Equation (5):

(5) $$Q_{wrist/effector}\;=\;\left[\begin{array}{cccc} 1 & 0 & 0 & 0\\ 0 & 1 & 0 & 0\\ 0 & 0 & 1 & O_{Z}\\ 0 & 0 & 0 & 1 \end{array}\right]\left[\begin{array}{cccc} \cos\alpha & 0 & \sin\alpha & 0\\ \sin\beta \sin\alpha & \cos\beta & -\sin\beta \cos\alpha & 0\\ -\cos\beta \sin\alpha & \sin\beta & \cos\beta \cos\alpha & T_{Z}\\ 0 & 0 & 0 & 1 \end{array}\right]$$

where β = atan2(D−BW), α = atan2(A−CW), as defined in Equations (2) and (3), O_Z_ is the fixed offset distance between the origin of the reference frame associated with the sensors and the reference frame assigned to the robot end-effector’s point of attachment, and T_Z_ is defined in Equation (4). A similar equation can be obtained for the external sensor array. This is achieved by substituting A, B, C and D with E, F, G and H, respectively for the estimates of β and α in Equation (5).

## 5. Compliant Wrist Experimental Characterization and Performance Evaluation

The designed compliant wrist was submitted to a comprehensive series of tests [31], including a detailed characterization of the response of the embedded IR range sensors, the impact of the calibration procedure, as well application experiments conducted with the compliant wrist mounted on a physical industrial manipulator while executing surface following procedures for particles collection. This section details the results of some of these experiments.

### 5.1. Range Sensor Layers Characterization

**Figure 14:**
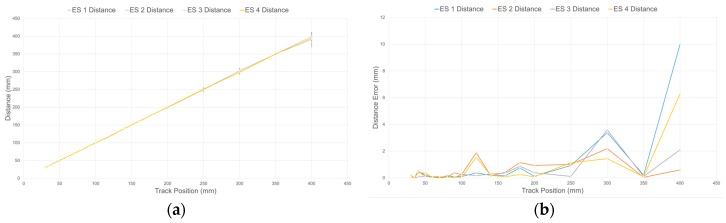
(**a**) Calibrated output of IR range sensors over range of operation and standard deviation on specific calibration points, and (**b**) distance error over range of operation.

**Figure 15:**
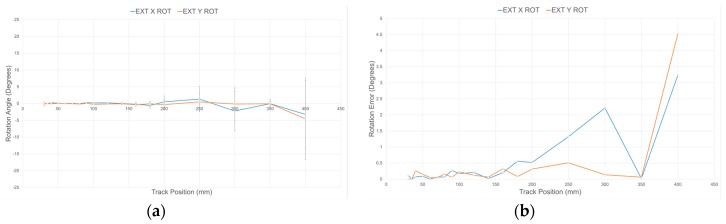
(**a**) Rotational deviation with respect to normal orientation of calibration board (0°) for calibrated IR range sensors over range of operation and standard deviation on specific calibration points; and (**b**) error on rotation over range of operation.

Using the embedded moving average filters with a window length of 33 samples and the calibration procedure detailed in Section 4.3, the response of each sensory layer implemented on the compliant wrist was characterized. Figure 14a shows the estimated distance for four IR range sensors after calibration over the entire range of operation of the compliant wrist, against the actual ground truth distance, as measured by the encoders installed on the calibration motorized track. Error bars, representing the standard deviation, are also depicted at the location of each of the 18 specific calibration points used in the process, and the remaining error is depicted in Figure 14b. The result demonstrate that, independently from the sensor considered, high precision is achieved at closer range, while the output tends to fluctuate slightly more as distance increases. It was determined experimentally that the average resolution of individual IR range sensors is 0.085 mm within a depth of 5 cm, and 2.3 mm at a depth of 40 cm. These characteristics compare advantageously with the performance of other compliant wrist systems reported in the literature [25,27]. Similarly, Figure 15 shows the mean angular deviation and the corresponding error, about the X and Y axes respectively, from the normal orientation (that is 0°) of the calibration board mounted on the motorized track, as captured with calibrated sensors. For orientation estimates, the average angular sensitivity is about 0.5° at shortest distance and up to about 1.4 degree over the largest range of operation. While the manufacturer’s IR sensors specification sheets indicate a suitable range of operation from 40 mm to 300 mm, our characterization and calibration process covered a broader range, from 30 mm to 400 mm, as shown in Figure 14 and Figure 15. After having closely studied the individual response of all sensors, an operational range of 30 to 300 mm was selected, based on the reliability of the calibration achieved over that range.

**Figure 16:**
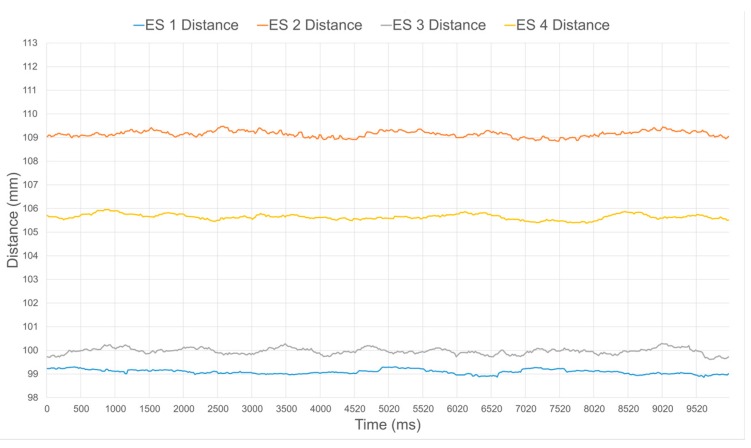
Uncalibrated distance estimates from four IR range sensors for ground truth distance of a surface located 90 mm in front of the compliant wrist.

**Figure 17:**
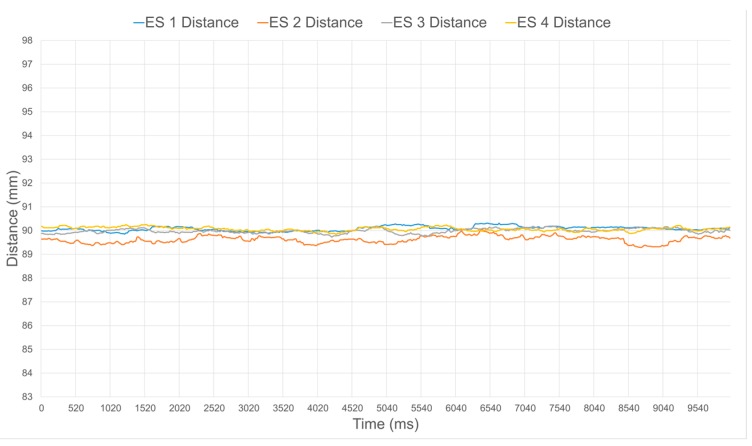
Calibrated distance estimates from four IR range sensors for ground truth distance of a surface located 90 mm in front of the compliant wrist.

Figure 16 and Figure 17 illustrate the distance estimates from four different sensors, before and after calibration respectively, when operating at a ground truth distance of 90 mm. Without calibration, larger disparities in estimated distance in between the sensors clearly appear, as seen in Figure 16. The distance is also systematically over estimated due to the nonlinear response of the sensors and the inherent bias in their response when compared to the specified value, as can be easily observed in Figure 8 at 90 mm. Over the 10 s of sampling considered, which represents about 500 samples, the average and standard deviation values on the distance for each individual sensor are summarized in Table 1. Once the calibration procedure is applied, all sensors produce a much more accurate and stable estimate, as shown in Figure 17 and Table 1.

**Figure 18:**
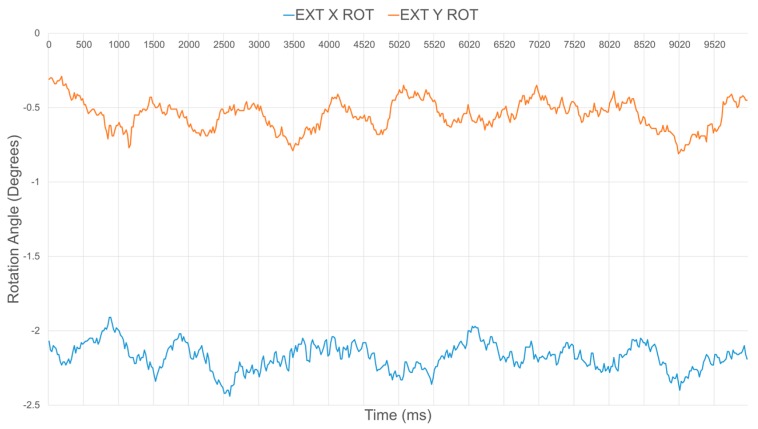
Uncalibrated orientation estimates for ground truth relative orientation of a surface with 0° rotation about both the X and Y axes.

**Figure 19:**
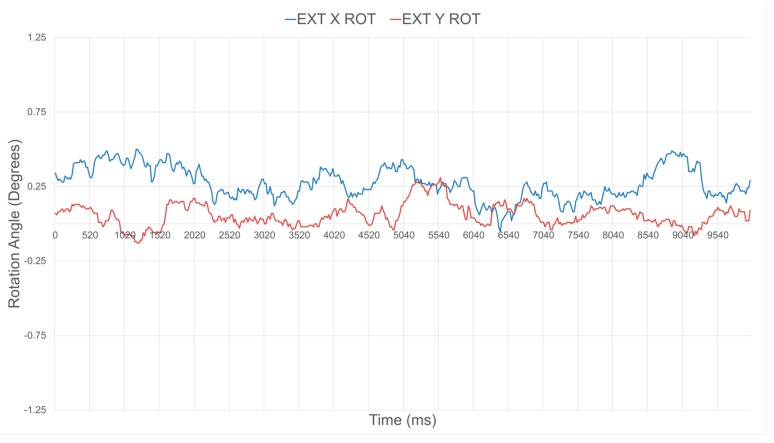
Calibrated orientation estimates for ground truth relative orientation of a surface with 0° rotation about both the X and Y axes.

By ensuring that all sensors are producing accurate distance outputs, rotation estimates around the X and Y axes are also significantly improved. Figure 18 and Figure 19 depict the relative estimated surface orientation, before and after calibration respectively, of a planar surface placed parallel to the sensor’s base plate. The expected ground truth rotation value about either axis is therefore 0°. Table 2 summarizes the average and standard deviation values over about 500 samples on the orientation about the X and Y axes of the compliant wrist under these two operational conditions. Rotation estimates tend to be slightly more variable than the distance estimates, but the benefits of the proposed rigorous calibration stage involved during construction of the compliant wrist are clearly demonstrated.

### 5.2. Compliant Wrist Performance Experimental Evaluation

**Figure 20:**
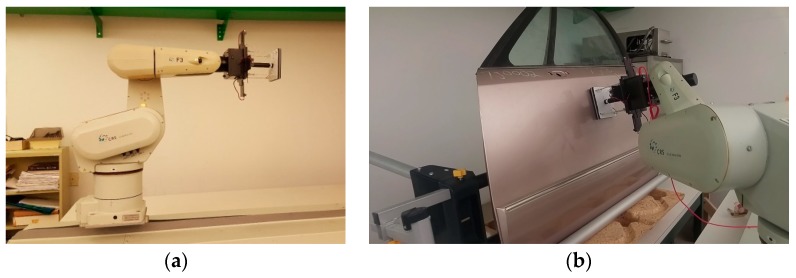
Experimental performance evaluation of the compliant wrist: (**a**) mounted as the end-effector of a CRS-F3 manipulator and embedded instrumentation integrated in the robot control loop; and (**b**) performing surface following over various surfaces.

**Figure 21:**
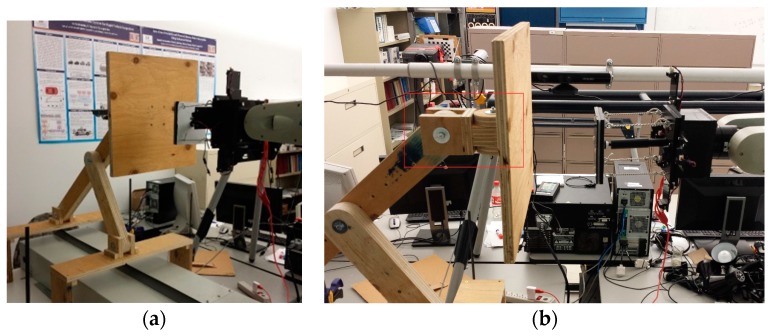
Compliant wrist operating in proximity to a planar surface: (**a**) general configuration; and (**b**) side view evidencing the prescribed gap in between the compliant plate and the surface.

**Figure 22:**
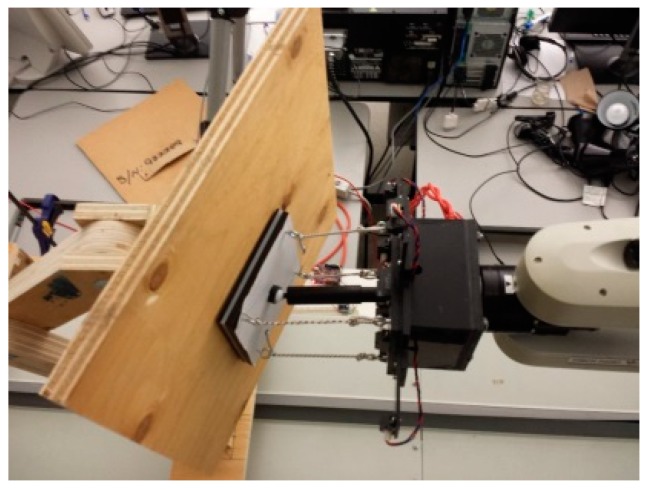
Compliant wrist operating in contact mode while physically interacting with an angled planar surface.

To further evaluate the performance of the compliant wrist under various operating conditions, it was mounted onto a CRS-F3 6 degrees-of-freedom manipulator robot, as shown in Figure 20a. Given the main application considered for this research, the testing protocol consisted of integrating the compliant wrist in the control loop of the manipulator to collect measurements during an approach and a contact phases, respectively, allowing the manipulator to interact with a surface under inspection, while providing evaluation on the accuracy of the overall system [30]. While surfaces of various shapes, including automotive body panels, were processed with success, as shown in Figure 20b, a planar wooden surface was mainly used to conduct the experiments reported in this paper, in order to ensure accurate determination of the compliant wrist capabilities. Two main interaction stages were examined: (*i*) cases where the wrist is positioned at a particular orientation and distance away from the surface, as shown in Figure 21, that is when the external sensory layer measures the distance to a proximal surface; and (*ii*) cases where the compliant surface of the wrist is in contact with a planar surface under various orientations, as shown in Figure 22, that is when the internal sensory layer monitors the relative transformation between the base and the compliant plates of the wrist. A special mount was fabricated to securely hold a planar surface in a preset orientation that can be accurately measured, while the robot equipped with the compliant wrist could effectively approach, and then come into contact with the surface, which would remain static. The robot is programmed to automatically reorient its end-effector tool plate to maintain a preset orientation with respect to the detected planar surface, in response to the measurements collected in real time by the compliant wrist, either in the proximity or the in-contact mode of operation. In proximity mode, the end-effector must also preserve a preset distance to the planar surface, while in contact mode, the manipulator must match the position of the compliant plate with that of the planar surface. In all test cases, distance measurements were also recorded and subsequently compared to ground truth values obtained by manual distance and orientation measurements over the planar surface. These experiments provided data to evaluate the stability and accuracy of the pose estimates provided by the instrumented compliant wrist, under proximity and in-contact operational conditions. The response time of the compliant wrist is mainly limited by the speed of the sensors and the filtering applied to the input signals. However, in these experiments the system was not specifically characterized in terms of a step response, mainly because the collection of experimental data was performed at only 50 Hz given the bottleneck caused by writing speeds to an SD card that was used to record the data from the sensors. In normal operational conditions, where data is only processed to control the manipulator but not recorded, faster response can be achieved while relying on real-time measurements. In spite of that, this section demonstrates the great stability of the wrist-equipped robot response to adapt to distance and orientation of an object, even with a lower sampling rate.

**Figure 23:**
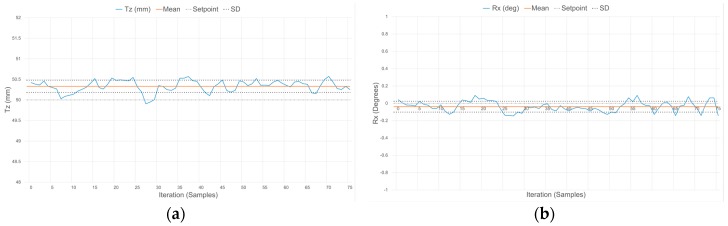
Response of the compliant wrist operating in proximity mode while measuring: (**a**) distance to; and (**b**) relative orientation of a fixed planar surface placed at 50 mm in front of the wrist and perpendicular to its main aiming direction (0°).

**Figure 24:**
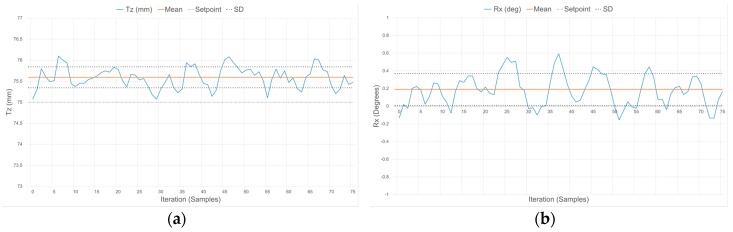
Response of the compliant wrist operating in proximity mode while measuring: (**a**) distance to; and (**b**) relative orientation of a fixed planar surface placed at 75 mm in front of the wrist and perpendicular to its main aiming direction (0°).

**Figure 25:**
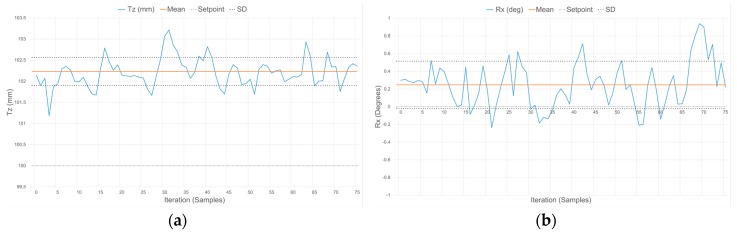
Response of the compliant wrist operating in proximity mode while measuring: (a) distance to; and (b) relative orientation of a fixed planar surface placed at 100 mm in front of the wrist and perpendicular to its main aiming direction (0°).

Figure 23, Figure 24 and Figure 25 demonstrate the characteristic response of the compliant wrist while measuring the distance and relative orientation of a planar surface located at various distances, respectively 50, 75 and 100 mm, in front of the compliant wrist (proximity mode of operation) and oriented perpendicular to the compliant wrist’s aiming direction. 75 data samples are collected over time for each distance, with the planar surface and compliant wrist remaining in static configurations, in order to evaluate the stability of the response. The results, which are further quantified in Table 3, exhibit some variations for both the translation and the rotation measurements over time, with a slight degradation as the distance increases. This is fully expected given the nature of the IR range sensor characteristic response, as discussed in Section 5.1.

To further monitor the influence of orientation on the compliant wrist measurements accuracy, Table 3 also reports on average distance and rotation estimates provided by the compliant wrist when operating in the proximity mode and for distances between the wrist and the target planar surface (T_z_) set to 50, 75 and 100 mm, but with a different relative target orientation of −30° around the X axis to be maintained. The measurements reported in Table 3 represent averaged values over the series of 75 samples collected while the manipulator is kept static in front of the fixed planar surface. The results confirm that the accuracy is not significantly impacted by the relative orientation of the planar surface with respect to the compliant wrist’s main orientation. Similar tests were conducted with a deflection about the Y axis and led to the same conclusion.

**Figure 26:**
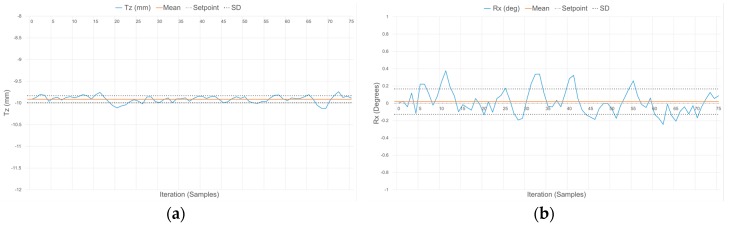
Response of the compliant wrist operating in contact mode while measuring: (**a**) distance to; and (**b**) relative orientation of the compliant plate matching that of a fixed planar surface, for a distance equivalent to −10 mm and a surface perpendicular to the wrist’s main aiming direction (0°).

Similarly, Table 4 reports on average distance and rotations estimates provided by the wrist when operating in the contact mode. In this case, two distances (T_z_) are considered, −10 and −20 mm, corresponding to the compression magnitude of the compliant wrist under the force exerted by the surface with which it is in contact. Respective rotations of the planar surface with respect to the wrist are 0° (parallel) and −10° (angled). The signal variations in distance and orientation are fairly similar for both compression distances. The smaller distances allowed by the wrist when in contact with a surface generate a stable response and impose lower limits to the errors associated with the parameters, given that the precision of IR sensors was demonstrated to be higher at closer range. This is also reflected in the stability of the internal sensors layer, as depicted in Figure 26 for the case where the compression distance is −10 mm and the orientation of the compliant plate matching the planar surface is 0°.

In both operation modes, the slight deviations of the mean measured values from the ground truth distance and orientation are due in part to the signal acquisition and processing performed on the embedded microcontroller being restricted to a 10-bit quantization, to the degree of accuracy with which the calibration of the IR range sensors can effectively be performed, as well as the difficulties faced when trying to obtain sub-millimetric precision on the ground truth values that measurements are compared with. This results in slightly reduced accuracy in the measurements although precision is maintained, as evident by the narrow standard deviation (SD) boundaries shown in Figure 23, Figure 24, Figure 25 and Figure 26, which indicate that stable signals are obtained in all circumstances. Overall, these experiments demonstrate the accuracy and stability achieved by the instrumentation embedded in the compliant wrist.

With a resolution varying from 0.085 mm and 0.5 degree for closest range, and up to 2.3 mm and 1.4° over the largest operational range, the prototype compliant wrist compares well with worst-case accuracies of 0.6 mm for translation and 0.0099 radians (0.57°) for rotation reported in [25]. The compliant plate of the prototype also supports a translation range of −25 mm to +10 mm with rotation ranges for both the X and Y axes of ±40°. Comparatively, [27] reports a 10 mm travel distance of the upper plate. The developed compliant wrist is therefore more versatile than and as accurate as previous designs reported in the literature, while adding this extra sensing layer that allows to provide real-time feedback to the robot controller before establishing contact with the surface, an important feature that did not exist in previous designs.

Experiments demonstrated that precision increases as objects are brought closer to the embedded sensors, thereby improving pose estimates generated by the compliant wrist as it approaches objects. With the proposed calibration procedure being able to produce uniform sensor outputs whose accuracy is within acceptable tolerance for a large range of applications, the compliance provided by the mechanical structure of the wrist also affords extra immunity to limited measurements precision during motion. In the event that there exists unwanted discrepancies between the actual pose of the objects to interact with and the pose reported by the compliant wrist, the compliance still allows for safe interaction with objects when contact occurs, offering a relative level of protection through its own compression, both for the contacted object and the robot structure, against sudden increases in contact forces.

## 6. Conclusions

This paper presented the design and extensive experimental characterization and validation of a flexible and affordable compliant mechanical structure designed and equipped with embedded range sensors and signal processing to support multi-step and multi-modal robotic interaction between a manipulator and objects located in its environment. The target application considered was that of surface following to achieve particles collection in the context of automobile vehicles screening, but the proposed compliant wrist and sensing device can be beneficial in a multiplicity of robotic scenarios. Beyond its demonstrated compliance and pose estimation accuracy, an important characteristic of the device is that it is stand-alone and not designed for the specific characteristics of a manipulator robot. For that reason, it can easily be mounted on and integrated in the control loop of any industrial robot. The entirely embedded signal processing stage and wireless communication capability are meant to easily interface with controller programming languages of any generation of robots. The pose estimation measurements provided by the external array of IR range sensors allow for real-time refinement of the trajectory while a manipulator is approaching a surface to ensure smooth initial contact. That is, the instrumented compliant wrist provides live input to the robot controller to closely monitor the interaction with an object while the pre-planned trajectory is executed, and permits slight corrections to this trajectory to be performed in order to best adapt the configuration of the end-effector with the surface of the object. This functionality is provided as long as the end-effector remains within the operational range of the IR sensors embedded on the compliant wrist with respect to the considered surface. This is an original and important feature that was never considered in previous designs of compliant wrists reported in the literature. Information from the internal sensory layer is later used to control the robot’s motion after contact with the surface is established, while the mechanism provides a level of compliance to the manipulator that would otherwise act as a rigid structure. Experimental validation of the compliant wrist mounted on an industrial manipulator demonstrated that the compliant wrist system is capable of achieving precise real-time measurements, reaching sub-millimeter precision at closer range. Additionally, the physical compliance afforded by the compliant wrist prevents large impact forces to occur during non-contact to contact transitions in between the manipulator’s end-effector and any surface submitted to interaction.

## Figures and Tables

**Table 1:** Average value and standard deviation over 500 samples of the distances from four individuals IR range sensors embedded in the compliant wrist, without and with calibration, and ground truth distance of 90 mm.

	Sensor 1 (mm)	Sensor 2 (mm)	Sensor 3 (mm)	Sensor 4 (mm)
Without calibration	99.09 ± 0.10	109.15 ± 0.13	99.97 ± 0.13	105.65 ± 0.12
With calibration	90.08 ± 0.10	89.62 ± 0.15	89.98 ± 0.10	90.07 ± 0.08

**Table 2:** Average value and standard deviation on the surface orientation relative to the compliant wrist, without and with calibration, and ground truth orientation of 0° about both axes.

	Rotation about X (°)	Rotation about Y (°)
Without calibration	−2.17 ± 0.09	−0.54 ± 0.10
With calibration	0.27 ± 0.11	0.06 ± 0.08

**Table 3:** 3-DOF transformation parameters measured when operating at three different distances to a planar surface, parallel to the compliant wrist (0°), or with a −30° rotation about X.

	Without Relative Rotation (R_x_ = 0°; R_y_ = 0°)			With Set Rotation of −30° (R_x_ = −30°; R_y_ = 0°)		
Set Distance	Measured T_z_ (mm)	Measured R_x_ (°)	Measured R_y_ (°)	Measured T_z_ (mm)	Measured R_x_ (°)	Measured R_y_ (°)
T_z_ = 50 mm	50.33 ± 0.15	−0.04 ± 0.06	0.01 ± 0.12	49.32 ± 0.49	−30.62 ± 0.33	−0.16 ± 0.04
T_z_ = 75 mm	75.59 ± 0.25	0.19 ± 0.18	0.06 ± 0.24	75.47 ± 0.74	−29.97 ± 0.40	0.09 ± 0.19
T_z_ = 100 mm	102.23 ± 0.33	0.25 ± 0.27	−0.06 ± 0.22	101.60 ± 0.62	−30.47 ± 0.33	0.38 ± 0.22

**Table 4:** 3-DOF transformation parameters measured when operating at two different levels of compression of the compliant plate in contact with a planar surface that is either parallel to the compliant wrist (0°), or angled with a −10° rotation about X.

	Without Relative Rotation (R_x_ = 0°; R_y_ = 0°)			With Set Rotation of −10° (R_x_ = −10°; R_y_ = 0°)		
Set Distance	Measured T_z_ (mm)	Measured R_x_ (°)	Measured R_y_ (°)	Measured T_z_ (mm)	Measured R_x_ (°)	Measured R_y_ (°)
T_z_ = −10 mm	−9.91 ± 0.08	0.02 ± 0.14	0.05 ± 0.15	−9.88 ± 0.09	−10.36 ± 0.13	−0.10 ± 0.19
T_z_ = −20 mm	−19.91 ± 0.07	−0.08 ± 0.08	−0.01 ± 0.08	−20.01 ± 0.07	−10.21 ± 0.11	0.13 ± 0.14

## References

[1] Laferrière P., Payeur P. Proximity and Contact Sensing with Instrumented Compliant Wrist for Close Guicance of Robotic Manipulators. Proceedings of the 3rd International Electronic Conference on Sensors and Applications, Sciforum Electronic Conference Series.

[2] Quigley M., Asbeck A., Ng A. A Low-Cost Compliant 7-DOF Robotics Manipulator. Proceedings of the IEEE International Conference on Robotics and Automation.

[3] Pratt G.A., Williamson M.M. Series of Elastic Actuators. Proceedings of the IEEE/RSJ International Conference on Intelligent Robots and Systems—Human Robot Interaction and Cooperative Robots.

[4] Bach-y-Rita P., Kercel S.W. (2003). Sensory Substitution and the Human-Machine Interface. Trends Cognit. Sci..

[5] Torres-Jara E. Obrero: A Platform for Sensitive Manipulation. Proceedings of the IEEE-RAS International Conference on Humanoid Robotics.

[6] Bicchi A., Peshkin M.A., Colgate J.E. (2008). Safety for Physical Human-Robot Interaction. Springer Handbook of Robotics.

[7] Siciliano B., Villani L. (1999). Robot Force Control.

[8] Siciliano B., Sciavico L., Villani L., Oriolo J. (2009). Robotics: Modelling, Planning and Control.

[9] Li E.C., Li Z.M. (2011). Surface Tracking with Robot Force Control in Unknown Environment. Adv. Mater. Res..

[10] Ibrayev R., Jia Y.B. (2012). Recognition of Curved Surfaces from “One-dimensional” Tactile Data. IEEE Trans. Autom. Sci. Eng..

[11] Payeur P., Pasca C., Cretu A.-M., Petriu E.M. (2005). Intelligent Haptic Sensor System for Robotic Manipulation. IEEE Trans. Instrum. Measurem..

[12] Hutchinson S., Hager G., Corke P. (1996). A tutorial on visual servo control. IEEE Trans. Robot. Autom..

[13] Hosoda K., Igarashi K., Asada M. Adaptive Hybrid Visual Servoing/Force Control in Unknown Environment. Proceedings of the International Conference on Intelligent Robots and Systems.

[14] Siradjuddin I., Behera L., McGinnity T.M., Coleman S. A position based visual tracking system for a 7 DOF robot manipulator using a Kinect camera. Proceedings of the 2012 International Joint Conference on Neural Networks.

[15] García G.J., Gil P., Llácer D., Torres F. Guidance of Robot Arms using Depth Data from RGB-D Camera. Proceedings of the 10th International Conference on Informatics in Control, Automation and Robotics.

[16] Castano A., Hutchinson S. (1994). Visual Compliance: Task-directed Visual Servo Control. IEEE Trans. Robot. Autom..

[17] Fisher W.D., Mujtaba M.S. (1992). Hybrid Position/Force Control: A Correct Formulation. Int. J. Robot. Res..

[18] Fomena R.T., Quintero C.P., Gridseth M., Jagersand M. Towards Practical Visual Servoing in Robotics. Proceedings of the 10th Conference on Computer and Robot Vision.

[19] Cutkosky M.R., Wright P.K. Position Sensing Wrists for Industrial Manipulators. Proceedings of the 12th International Symposium on Industrial Robotics.

[20] Wolffenbuttel R.F., Mahmoud K.M., Regtien P.P.L. (1990). Compliant Capacitive Wrist Sensor for Use in Industrial Robots. IEEE Trans. Instrum. Measurem..

[21] Wolffenbuttel R.F., Mahmoud K.M., Regtien P.P.L. Multiaxis Compliant Capacitive Wrist Sensor for Use in Automated Assembly with Industrial Robots. Proceedings of the IEEE International Conference on Instrumentation and Measurement Technology.

[22] Choi S.W., Choi Y.J., Kim S. Using a Compliant Wrist for a Teleoperated Robot. Proceedings of the IEEE/RSJ International Conference on Intelligent Robots and Systems.

[23] Hashimoto M., Imanura Y. Design and Characteristics of a Parallel Link Compliant Wrist. Proceedings of the IEEE International Conference on Robotics and Automation.

[24] Sergi F., Lee M.M., O’Malley M.K. Design of a Series Elastic Actuator for a Compliant Parallel Wrist Rehabilitation Robot. Proceedings of the IEEE International Conference on Rehabilitation Robotics.

[25] Lindsay T.S., Sinha P.R., Paul R.P. An Instrumented Compliant Wrist for Robotics Applications. Proceedings of the IEEE International Conference on Robotics and Automation.

[26] Sinha P.R., Xu Y., Bajcsy R.K., Paul R.P. (1993). Robotic Exploration of Surfaces with a Compliant Wrist Sensor. Int. J. Robot. Res..

[27] Petriu E., McMath W.S., Yueng S.S.K., Trif N. (1992). Active Tactile Perception of Object Surface Geometric Profiles. IEEE Trans. Instrum. Measurem..

[28] Fareh R., Payeur P., Nakhaeinia D., Macknojia R., Chavez-Aragon A., Cretu A.-M., Laferrière P., Laganière R., Toledo R. An Integrated Vision-Guided Robotic System for Rapid Vehicle Inspection. Proceedings of the IEEE Intl Systems Conference.

[29] Chavez-Aragon A., Macknojia R., Payeur P., Laganière R. (2013). Rapid 3D Modeling and Parts Recognition on Automotive Vehicles using a Network of RGB-D Sensors for Robot Guidance. J. Sens..

[30] Nakhaeinia D., Laferrière P., Payeur P., Laganière R. Safe Close-Proximity and Physical Human-Robot Interaction Using Industrial Robots. Proceedings of the 12th Conference on Computer and Robot Vision.

[31] Laferrière P. (2016). Instrumented Compliant Wrist System for Enhanced Robotic Interaction. Master’s Thesis.

